# Low cost air quality sensors “PurpleAir” calibration and inter-calibration dataset in the context of Beirut, Lebanon

**DOI:** 10.1016/j.dib.2022.108008

**Published:** 2022-03-01

**Authors:** Nareg Karaoghlanian, Batoul Noureddine, Najat Saliba, Alan Shihadeh, Issam Lakkis

**Affiliations:** a Department of Mechanical Engineering, American University of Beirut, Lebanon; b Department of Chemistry, American University of Beirut, Lebanon

**Keywords:** Low-cost sensor, Particulate matter, PM10, PM2.5, PurpleAir, Calibration, Inter-sensor variability, Beirut

## Abstract

The PurpleAir PA-II-SD is a low-cost particulate matter (PM2.5 and PM10) sensor that is currently available on the market. It is one of many such low-cost and commercially available particulate matter sensors which are being adopted by individuals and researchers worldwide. With growing use of these sensors, there is an interest in better understanding the performance and characteristics of these devices.

Data was collected from twelve of these low-cost PurpleAir PA-II-SD sensors and two high fidelity Met One E-BAM PLUS instruments installed at a single location, on the campus of the American University of Beirut, in Beirut, Lebanon over a period of time from June 28, 2020 to September 30, 2020. The data was collected with the aim of assessing inter-sensor variability for the PurpleAir sensors and the sensor accuracy of the PurpleAir when compared to a high fidelity Met One E-BAM PLUS instrument.

## Specifications Table

**Table:** 

Subject	Environmental science-pollution
Specific subject area	Air pollution sensor calibration
Type of data	Table Graph
How data were acquired	Instruments: • 12 Purple Air PA-II-SD. • 1 Met-One E-BAM PLUS configured for measuring PM2.5. • 1 Met-One E-BAM PLUS configured for measuring PM10.
Data format	• Raw. • Analysed.
Parameters for data collection	• Data logging by PurpleAir instruments was every 2 m. • Data logging by E-BAM Plus is recorded every 1 h. • Data was retimed and averaged to an hourly reading to synchronize with the reference E-BAM PLUS data. • Negative readings were excluded (2 datapoints out of 2163). • Exclusion of timestamps with missing data points for any sensor for that timestamp.
Description of data collection	• PurpleAir are real-time optical air quality sensors measuring PM2.5 and PM10. • PurpleAir instruments have two optical sensors, channel A and B. Unless specified, data reported by a given instrument is the average of both channels.
Data source location	Institution: American University of Beirut City/Town/Region: Beirut Country: Lebanon Latitude and longitude for collected samples/data: 33.9N, 35.5E
Data accessibility	Repository name: Mendeley Data Data identification number: 10.17632/rh2z7s7btj.1 Direct link to the dataset: http://dx.doi.org/10.17632/rh2z7s7btj.1

## Value of the Data

• The data contains simultaneous measurements of twelve PurpleAir sensors [1] and two high-fidelity E-BAM PLUS instruments [2] measuring PM2.5 and PM10 levels in Beirut and allows for the assessment of inter-sensor variability between PurpleAir sensors and their accuracy compared to a high fidelity instrument.
• The data from the twelve PurpleAir sensors can enable PurpleAir users to quantify errors when comparing data from multiple sensors installed over a large area.
• The calibration coefficients reported here can enable PurpleAir sensor users to improve the accuracy of their PurpleAir measurements.
• The placement of the PurpleAir and Met One E-BAM PLUS sensors at the campus of the American University of Beirut provide measurements of the background PM levels within and around the city of Beirut over the time period from July 1, 2020 to September 30, 2020.
• The data provided here is a resource to allow the more than 20,000 users [5] (individuals, researchers, and weather forecasting agencies reporting air quality index [6]) to improve the accuracy of reporting of their data.

## 1 Data Description

The first part of the data summarizes the results of the linear regression of data from the PurpleAir sensor against the reference E-BAM PLUS instruments.

**Fig. 1:**
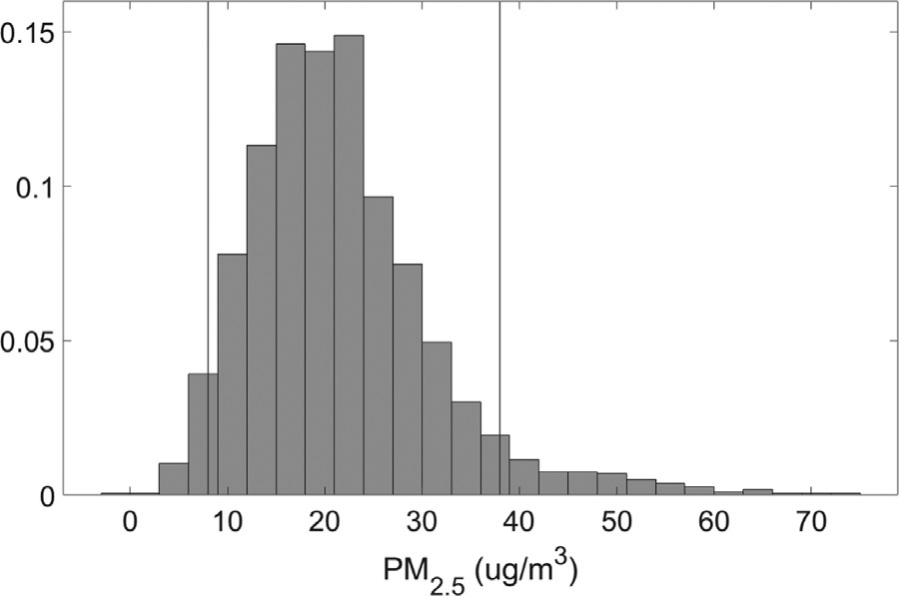
Normalized PDF for PM_2.5_ concentration. mean PM_2.5_ = 21.06 ug/m^3^, median PM_2.5_ = 20 ug/m^3^, 90% quantile: 8–38 ug/m^3^.

**Fig. 2:**
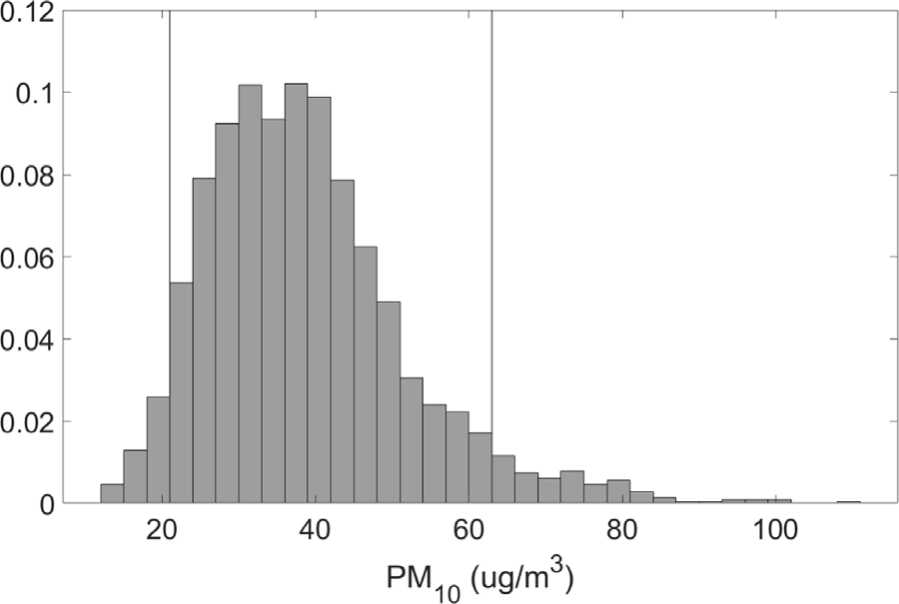
Normalized PDF for PM_10_ concentrations. mean PM_10_ = 38.25 ug/m^3^ median PM_10_ = 37 ug/m^3^, 90% quantile: 21–63 ug/m^3^.
• Table 2 is a summary of the regression coefficients which is shown in more detail in Figs. 3 and 4.

• Table 1 summarizes the errors (root mean square) for the two calibration and validation scenarios for the two PM ranges. The smaller range being selected from the 90% quantile of the concentration measurements shown in Figs. 1 and 2.

**Table 1:** Summary of calibration.

Scenario	RMSE
PM2.5 Calibration	4.4363
PM2.5 Validation	4.5463
PM2.5 Calibration (90% quantile)	3.9776
PM2.5 Validation (90% quantile)	4.2154
PM10 Calibration	8.0981
PM10 Validation	9.792
PM10 Calibration (90% quantile)	6.7409
PM10 Validation (90% quantile)	6.924

**Table 2:** Summary of coefficients.

Scenario	Slope	Intercept
PM2.5 Full range	0.48875	5.3084
PM10 Full range	0.55284	19.2953
PM2.5 90% quantile range 8–38 ug/m^3^	0.42204	7.2829
PM10 90% quantile range 21–61 ug/m^3^	0.47268	21.6036

**Fig. 3:**
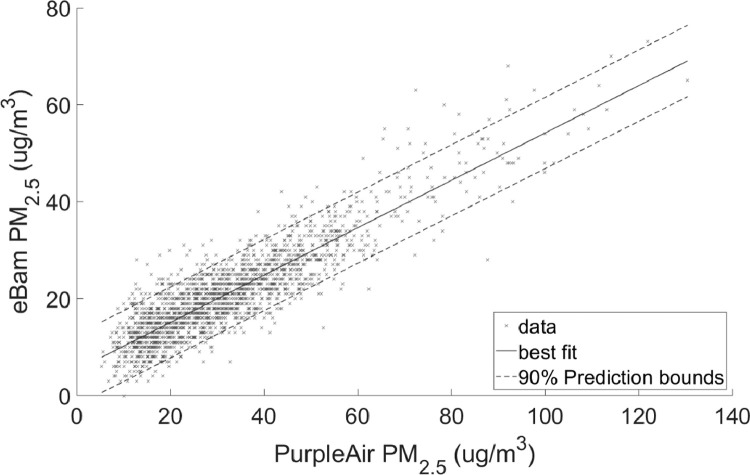
Linear regression with 90% prediction intervals for PM_2.5_ Regression coefficients: slope = 0.48875 and intercept = 5.3084 RMSE = 4.4363.

**Fig. 4:**
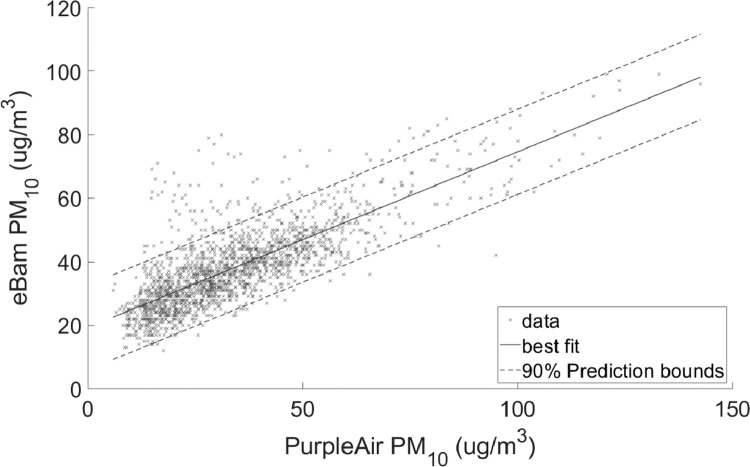
Linear regression with 90% prediction intervals for PM_10_ Regression coefficients: slope = 0.55284 and intercept = 19.2953 RMSE = 8.0981.
• Figs. 1 and 2, respectively, show the distributions of concentrations of PM_2.5_ and PM_10_ with the 90% quantile cut-off highlighted from the EBAM PLUS instruments; 8-38 ug/m^3^ for PM2.5 and 21-61 ug/m^3^ for PM10.
• Figs. 3 and 4 show the linear regression for PM2.5 and PM10, respectively, of the PurpleAir sensor against data from the EBAM PLUS instruments with 90% prediction intervals.
• Figs. 5 and 6 shows the result of the validation data set.

**Fig. 5:**
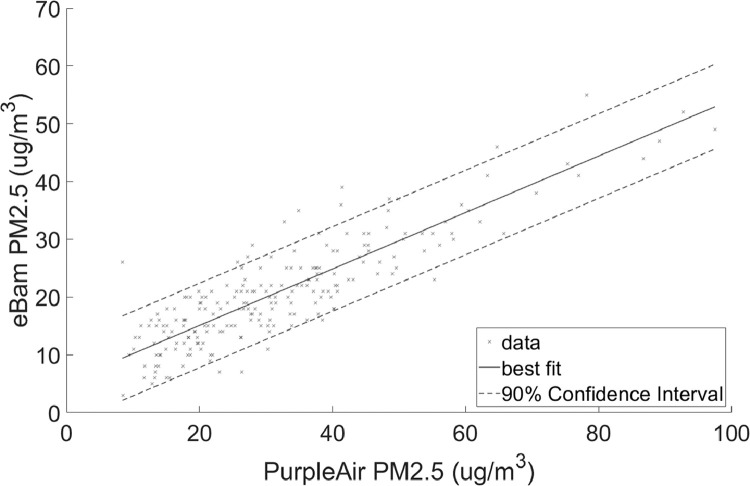
PM_2.5_ validation data with 90% confidence intervals RMSE = 4.5463.

**Fig. 6:**
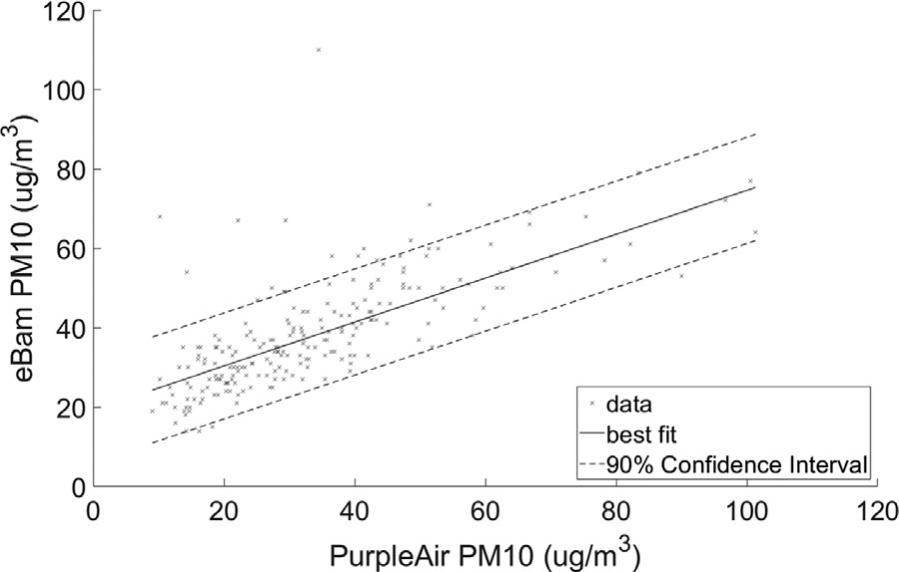
PM_10_ validation data with 90% confidence intervals. RMSE = 9.792.
• Fig. 7, Fig. 8, Fig. 9, Fig. 10 are similar to Fig. 3, Fig. 4, Fig. 5, Fig. 6 but regression is performed on the 90% quantile of each of the PM2.5 and PM10 concentrations.

**Fig. 7:**
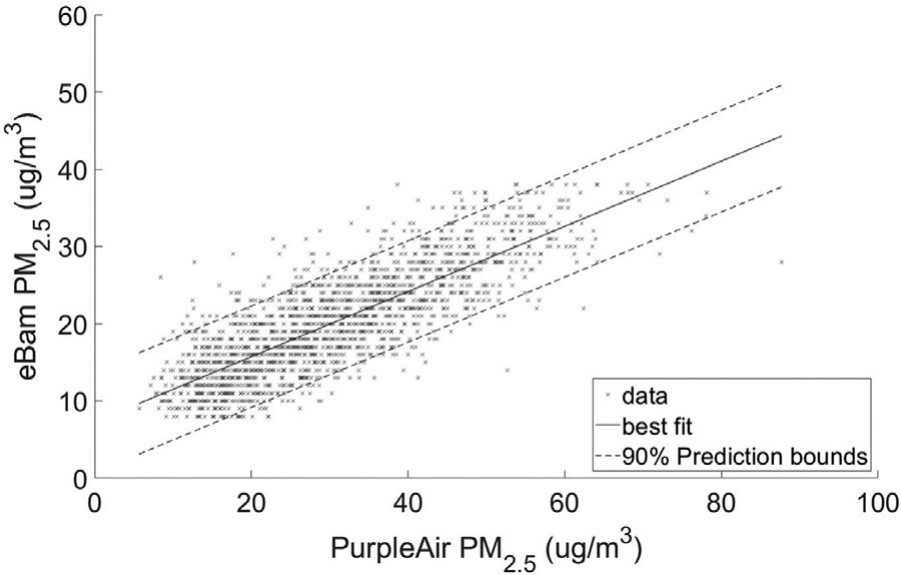
Linear regression with 90% prediction intervals for PM_2.5_ 90% quantile (8–38 ug/m^3^) Regression coefficients: slope = 0.42204 and intercept = 7.2829 RMSE = 3.9776.

**Fig. 8:**
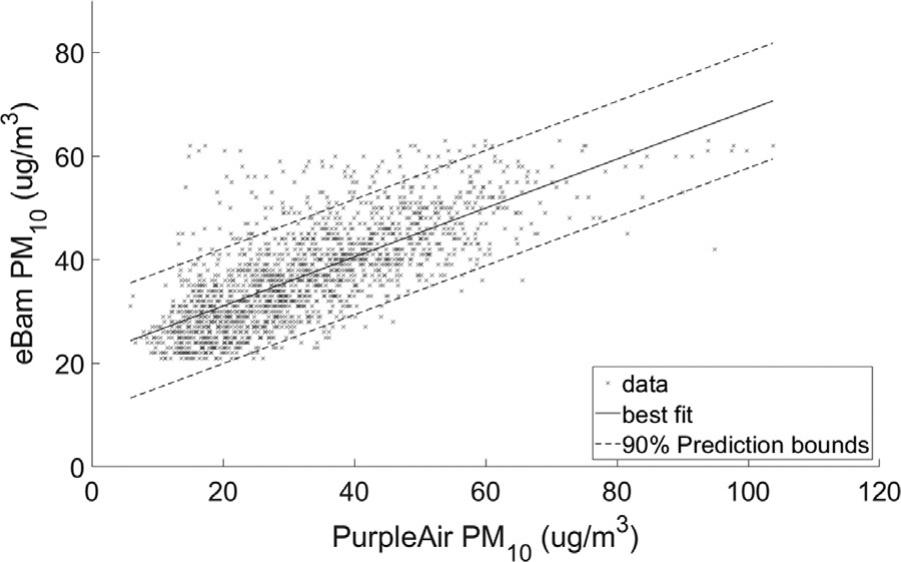
Linear regression with 90% prediction intervals for PM_10_ 90% quantile (21–63 ug/m^3^) Regression coefficients: slope = 0.47268 intercept = 21.6036 RMSE = 6.7409.

**Fig. 9:**
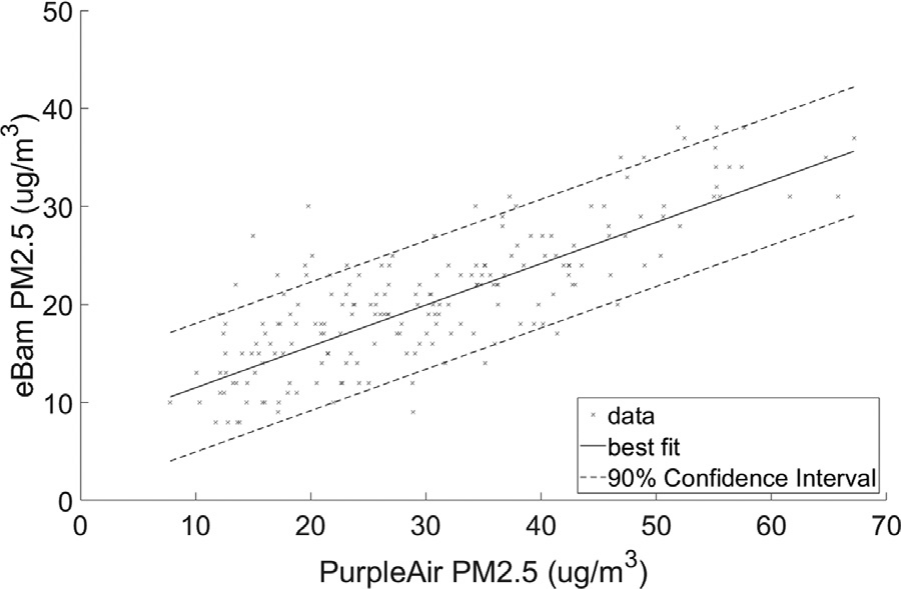
PM_2.5_ 90% quantile validation data with 90% confidence intervals RMSE = 4.2154.

**Fig. 10:**
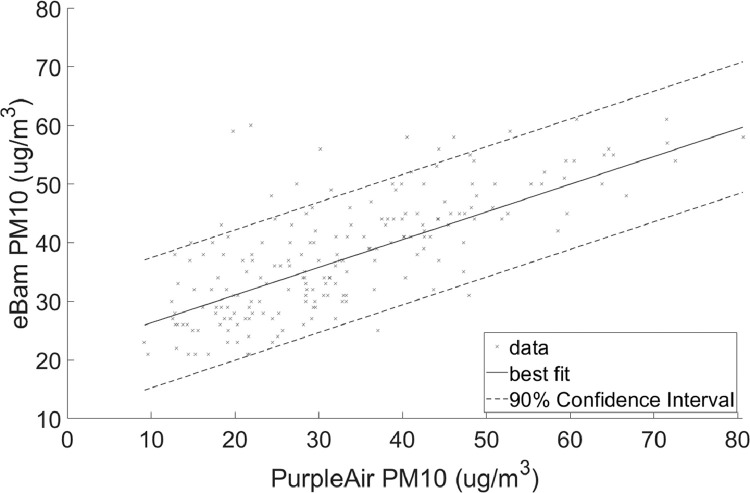
PM_10_ 90% quantile validation data with 90% confidence intervals. RMSE = 6.924.

The second part shows the data collected from 11 PurpleAir sensors placed at a single location to assess the precision of measurements between multiple sensors.

**Fig. 11:**
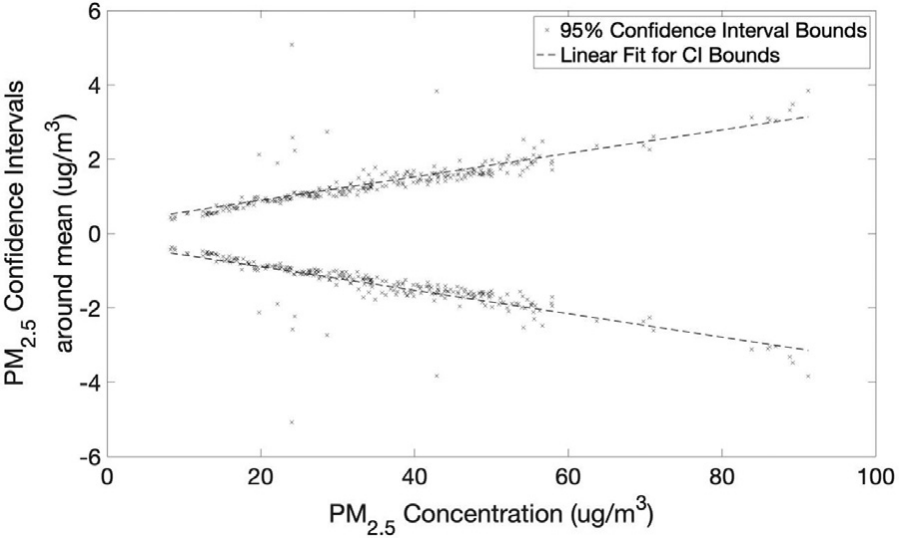
PM_2.5_ 95% confidence intervals around mean for 11 PurpleAir sensors with linear fit (slope +/-0.031542 and intercept +/-0.2639).

**Fig. 12:**
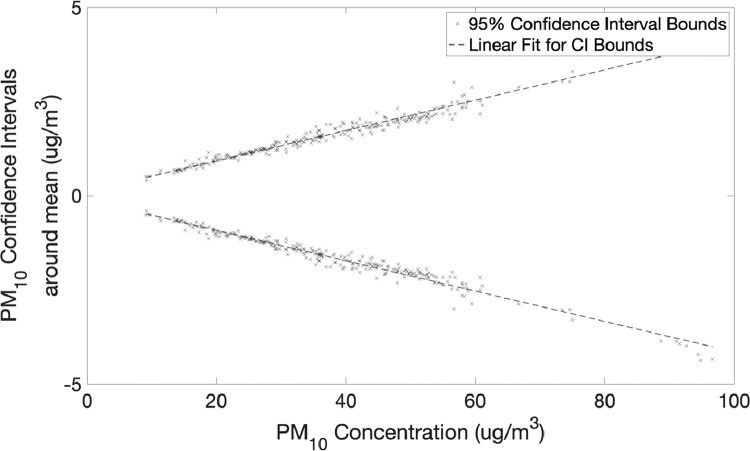
PM_10_ 95% confidence intervals around mean for 11 PurpleAir sensors with linear fit (slope +/-0.040278 and intercept +/-0.11654).

• Figs. 11 and 12 show 95% confidence interval around mean for PM2.5 and PM10 measurements from 11 PurpleAir sensors with the linear best fit.

The full dataset which is accessible at the repository is divided into two CSV files.

The file ‘MultiSensor_IntercalibrationData.csv contains hourly PM2.5 and PM10 data from eleven PurpleAir sensors for the date range between June 28, 2020 to July 11, 2020.

The columns are divided as such:

• (A): Time: Date and Time (Local Beirut Time).
• (B)-(AS): hourly average PurpleAir sensor data for PM2.5 and PM10 in units (ug/m3). Each individual sensor is identified by the prefix “S_X” where “X” represents the channel number. Each sensor has 4 columns: PM2.5A, PM2.5B, PM10A, PM10B where ‘A’ and ‘B’ represent the individual channels in each sensor.

The file ‘SingleSensor_CalibData.csv’ contains hourly PM2.5 and PM10 from a single PurpleAir sensor and two Met One EBAM-PLUS instruments for the date range from July 1, 2020 to September 30, 2020.

The file contains five columns:

• (A) Time_Beirut: Date and Time (Local Beirut Time).
• (B) PM2.5 ConHR (ug/m3): PM2.5 hourly concentration reading from MET ONE EBAM PLUS instrument.
• (C) PM10 ConHR (ug/m3): PM10 hourly concentration reading from MET ONE EBAM PLUS instrument.
• (D) meanAB_2_5: PM2.5 reading (ug/m3) of PurpleAir sensor. The value is the average of both channels A and B from the sensor.
• (E) meanAB_10: PM10 reading (ug/m3) of PurpleAir sensor. The value is the average of both channels A and B from the sensor.

## 2 Experimental Design, Materials and Methods

Data for ambient air pollution (PM2.5 and PM10) was collected on the campus of the American University of Beirut at 33.9N and 35.5E.

Twelve PurpleAir PA-II-SD sensors and two E-BAM PLUS were installed at a single location on the campus of the American University of Beirut. The dataset generated was used for a twofold purpose:

1. Generate a linear calibration curve for each of the PM2.5 and PM10 measurements of PurpleAir PA-II-SD sensors using Met One E-BAM PLUS instruments as reference (Table 2).
2. Report on the precision of measurements of PurpleAir sensors and inter-sensor variability by comparing measurements of multiple sensors from a single location (Figs. 11 and 12).

The location of the PurpleAir sensors was chosen to be the campus of the American University of Beirut which is located within the capital, Beirut, as it is representative of background PM levels within the city and also to have it adjacent to the Met One E-BAM PLUS instrument, the reference measurement, which are part of the American University of Beirut Air Pollution Observatory Project [3,4].

Data is reported by the E-BAM PLUS at an hourly interval and every two minutes for the PurpleAir sensors. These were averaged every hour to synchronize the reporting interval of all sensors.

For the calibration of the PurpleAir sensor against the E-BAM PLUS instrument, hourly data covering a span of three months (from July 1, 2020 to September 30, 2020) from a single PurpleAir sensor was used resulting in a total number of 2163 data points.

The dataset was split into two groups, the first for linear regression / curve fitting comprising 90% of the data points for the purpose of performing linear regression and the second comprising 10% of the data point for the purpose of validation of the curve fit.

For each size range (PM_2.5_ and PM_10_), two linear regressions were performed:

1) Using the entire span of concentrations in the dataset (Figs. 3 and 4).
2) Using the 90% quantile range of concentrations (Figs. 7 and 8).

The second regression (the 90% quantile range) is done for the purpose of achieving a better result for the regression with the outliers excluded. The improvement is apparent in a lower RMSE value for the regression for the 90% quantile when compared to the full range as seen in Table 1.

For assessing the inter-sensor variability eleven PurpleAir sensors were used, all located at a single site on the campus of the American University of Beirut with measurements covering a span of five weeks (from June 28, 2020 to July 09, 2020) for a total of 276 data points (hours) and the upper and lower bounds of the 95% confidence intervals were calculated across the span of measurements for this time period.

## CRediT authorship contribution statement

**Nareg Karaoghlanian:** Formal analysis, Data curation, Writing – original draft, Visualization. **Batoul Noureddine:** Investigation, Writing – review & editing. **Najat Saliba:** Resources, Data curation. **Alan Shihadeh:** Conceptualization, Writing – review & editing. **Issam Lakkis:** Supervision, Project administration, Conceptualization, Methodology, Writing – review & editing.

## Declaration of Competing Interest

The authors declare that they have no known competing financial interests or personal relationships which have or could be perceived to have influenced the work reported in this article.
